# Genetic variation in the tissue factor gene is associated with clinical outcome in severe sepsis patients

**DOI:** 10.1186/s13054-014-0631-9

**Published:** 2014-11-17

**Authors:** Dongwei Shi, Zhenju Song, Jun Yin, Mingming Xue, Chenling Yao, Zhan Sun, Mian Shao, Zhi Deng, Yaping Zhang, Zhengang Tao, Si Sun, Jin Zhang, Lingyu Xing, Zhimin Dong, Yuxin Wang, Chaoyang Tong

**Affiliations:** Department of Emergency Medicine, Zhongshan Hospital, Fudan University, 180 Fenglin Road, Shanghai, 200032 PR China

## Abstract

**Introduction:**

Activation of inflammation and coagulation was closely related and mutually interdependent in sepsis. Tissue factor (TF) and its endogenous inhibitor, tissue factor pathway inhibitor (TFPI) was the main regulators of the initiation of coagulation process. Altered plasma levels of TF and TFPI have been related to worse outcome in sepsis. The objective of this study was to investigate whether single nucleotide polymorphisms (SNPs) in the TF and TFPI genes were associated with risk and outcome for patients with severe sepsis.

**Methods:**

Seventeen SNPs in *TF* and *TFPI* were genotyped in samples of sepsis (*n* =577) and severe sepsis patients (*n* =476), and tested for association in this case–control collection. We then investigated correlation between the associated SNPs and the mRNA expression, and protein level of the corresponding gene. The mRNA levels of *TF* were determined using real-time quantitative reverse transcription-polymerase chain reaction and the soluble plasma levels of TF were measured using enzyme linked immunosorbent assay (ELISA) method.

**Results:**

Association analysis revealed that three *TF* SNPs in perfect linkage disequilibrium, rs1361600, rs3917615 and rs958587, were significantly associated with outcome of severe sepsis. G allele frequency of rs1361600 in survivor patients was significantly higher than that in nonsurvivor severe sepsis patients (*P* =4.91 × 10^-5^, odds ratio (OR) =0.48, 95% confidence interval (CI) 0.33 to 0.69). The association remained significant after adjustment for covariates in multiple logistic regression analysis and for multiple comparisons. Lipopolysaccharide-induced *TF*-mRNA expression levels in peripheral blood mononuclear cells from subjects carrying rs1361600 AG and GG genotypes, were significantly lower than those subjects carrying AA genotype (*P* =0.0012). Moreover, severe sepsis patients of GG and GA genotypes showed lower serum levels of TF than patients with AA genotype (*P*_adj_ =0.02). The plasma levels of TF were also associated with outcome of severe sepsis patients (*P*_adj_ =0.01). However, genotype and allele analyses did not show any significant difference between sepsis and severe sepsis patients.

**Conclusions:**

Our findings indicate that common genetic variation in *TF* was significantly associated with outcome of severe sepsis in Chinese Han population.

**Electronic supplementary material:**

The online version of this article (doi:10.1186/s13054-014-0631-9) contains supplementary material, which is available to authorized users.

## Introduction

Sepsis is an infection-initiated and inflammation-induced syndrome [1]. Despite progress in the development of antibiotics and other supportive care therapies, severe sepsis remains an unconquered challenge for clinicians with an unacceptable high mortality rate of 30 to 50% [2]. Although the pathogenesis of severe sepsis is not precisely understood, emerging evidence suggests that an exaggerated and uncontrolled systemic host inflammation and coagulation response to infectious pathogens leads to microvascular thrombosis, multiple organ dysfunction syndrome and death in severe sepsis [3].

Tissue factor (TF) and its endogenous inhibitor, tissue factor pathway inhibitor (TFPI), are the main regulators of the initiation of coagulation process [4,5]. The balance between TF and TFPI is essential in hemostasis and an alteration of this equilibrium may induce a procoagulant state. Recent studies indicated that the dysfunction of TF and TFPI was closely related to the severity and outcome of sepsis [6,7]. TF is not normally expressed by circulating blood cells or by vascular endothelial cells. During sepsis, a variety of stimuli such as proinflammatory cytokines and shear stress induce TF expression on monocytes and endothelial cells [8]. Factor VIIa binds to TF on the cell surface, forming TF/factor VIIa complexes. The complexes then bind to factor X, converting it to the activated form factor Xa, which leads to eventual thrombin formation and fibrin deposition [9-11]. Normally, thrombin generation via the TF pathway is controlled rapidly by TFPI. As the main regulator in the initial step of the coagulation cascade mediated by TF, TFPI binds to coagulation factors Xa and VIIa and TF and forms an inactive complex. It has been reported that the lower TFPI levels were strongly correlated with organ dysfunction as well as worse outcome of severe sepsis [6]. Furthermore, experimental studies proved the benefit of the early blockade of the TF–factor VIIa activated coagulation system in reducing both systemic and pulmonary inflammation as well as coagulation, and in improving lung physiology, histological results and even survival [12,13].

Sepsis is a complex syndrome resulting from the interactions between environmental factors and genetic factors. Clinical studies found that individuals varied significantly in their susceptibility to infection and in their ability to recover from apparently similar infectious processes. These differences can be partially explained by polymorphisms of the genes encoding proteins involved in mediating and controlling the innate immune response, the inflammatory cascade, coagulation and fibrinolysis [14]. Despite the prognostic value of hemostatic abnormalities in sepsis and the description of several hemostatic gene polymorphisms, only a few association studies sought to correlate these single nucleotide polymorphisms (SNPs) (mainly the factor V Leiden mutations and the 4G genotype in the promoter region of plasminogen activator inhibitor-1) with susceptibility to severe sepsis and disease severity [15-17]. In recent years, several polymorphisms in the promoter and coding region of the *TF* and *TFPI* genes have been identified, and some were associated with an increased risk of adverse outcomes in illnesses such as deep venous thrombosis, stroke and cardiovascular disease [18-21]. However, the relationship between genetic polymorphisms of *TF* and *TFPI* and survival in patients with severe sepsis has not been examined.

Given the potential association of an activated coagulation system with sepsis pathophysiology, particularly the role of the TF pathway as an important initiator of the coagulation system, *TF* and *TFPI* were excellent candidate genes for severe sepsis susceptibility. The objective of this study was thus to evaluate the association of *TF* and *TFPI* polymorphisms with the development and outcome of severe sepsis in the Chinese Han population. To gain further insight into the possible molecular basis of the association, we examined the functional influence *in vitro* of associated polymorphisms in the regulatory region of *TF*.

## Materials and methods

### Study design and enrollment

This study was approved by the Ethics Study Board of Zhongshan Hospital, Fudan University, Shanghai, China (record number 2006-23). Written informed consent was obtained from patients or the next of kin, carers or guardians on the behalf of the participants before enrollment. The diagnosis of sepsis, severe sepsis and septic shock was established according to the 2001 SCCM/ESICM/ACCP/ATS/SIS International Sepsis Definitions Conference [22] (Additional file 1). In this study, septic shock patients were classified as the severe sepsis group. Baseline characteristics (age, gender and previous health status) as well as clinical data including Acute Physiology and Chronic Health Evaluation II (APACHE II) and Sequential Organ Failure Assessment (SOFA) scores, source of infection, microbiology and ICU mortality were obtained after the patient met the sepsis criteria. The APACHE II and SOFA scores were calculated in the first 24 hours after the diagnosis of sepsis and severe sepsis. When cultures were absent or negative, the source of infection was determined by two senior physicians. All patients included in the study were followed up for 30 days or to hospital discharge. The discharged patients hospitalized for less than 30 days were followed up once a week by telephone until they were diagnosed at day 60. People that could not be contacted by our team worker were excluded from the study. Exclusion criteria included age below 18 years, pregnancy, severe chronic respiratory disease, severe chronic liver disease (defined as Child–Pugh score >10), malignancy, using of high-dose immunosuppressive therapy and AIDS diagnosis.

Recent analyses by genome-wide SNP variation showed that the central Han Chinese could be regarded as one single homogeneous population [23]. To reduce the potential confounding from ethnic backgrounds, we only enrolled people with self-reported origin of central Han Chinese, including indigenous people from Zhejiang Province, Jiangsu Province, Anhui Province and Shanghai City.

### Single nucleotide polymorphism selection and genotyping

Tag SNPs were selected based on the data of Han Chinese in Beijing from the HapMap project phase II. The tag SNPs covered the gene regions and up to 1 kb of 3′ as well as 5′ flanking regions of the candidate genes. Four tag SNPs for the 12.44 kb region encompassing the entire *TF* gene and eight tag SNPs for the 75.91 kb region encompassing the entire *TFPI* gene were identified by tagger within Haploview [24] using the following tagging criteria: pairwise tagging of the HapMap Han Chinese in Beijing population with *r*^2^ ≥ 0.8 and a minor allele frequency ≥5%. Additionally, five SNPs located within the gene region were also genotyped in this study as they showed association with deep venous thrombosis and ischemic arterial disease. A total of 17 SNPs were selected and genotyped in this study. The location and characterization of all selected SNPs are presented in Additional file 2.

Genomic DNA was extracted from whole blood with a FlexiGene DNA Kit (Qiagen, Hilden, Germany) in accordance with the protocol of the manufacturer. We genotyped these SNPs using the 12-plex GenomeLab SNPStream system (Beckman Coulter, California, USA). The primers for PCR and single base extension were designed with Beckman Coulter Autoprimer software and are presented in Additional file 3. Genotyping was carried out blind to case–control status. One duplicate sample was added to each 96-well sample plate for quality assurance and quality control validation of inter-plate discordance, and we placed an extra 10 duplicates into our sample set in order to test for experiment-wide discordance. The quality of the genotype data was assessed by testing for Hardy–Weinberg equilibrium using the chi-square distribution for each assay. The overall genotype call rate was 97%, and the accuracy was 99.96% according to duplicate analysis.

### Isolation and stimulation of cells from healthy subjects

To determine the associations between rs1361600 genotypes and *TF* mRNA levels in peripheral blood mononuclear cells (PBMCs), we investigated 45 subjects with the rs1361600AA genotype, 42 subjects with the GA genotype and three subjects with the GG genotype. All the subjects were healthy volunteers. PBMCs were derived using Ficoll gradient density centrifugation method. Isolated PBMCs were plated at a density of 1 × 10^6^ cells/ml in 24-well plates and cultured in RPMI 1640 medium with 10% fetal bovine serum at 37°C with 5% carbon dioxide. The cells were then incubated for 6 hours in the presence of 100 ng/ml *Escherichia coli* 0111:B4 lipopolysaccharide (LPS; Sigma, Saint Louis, USA).

### RNA purification and tissue factor mRNA expression analysis

Total RNA was extracted using the RNeasy Mini kit (Qiagen). RNA (100 ng) was used for cDNA synthesis using a High Capacity cDNA Reverse Transcription Kit (Applied Biosystems, Foster, USA) according to the manufacturer’s protocol. Quantitative RT-PCR was performed using SYBR Green (TaKaRa, Kyoto, Japan) on an ABI PRISM 7900 Sequence Detector (Applied Biosystems, Foster, USA) with SDS 2.1 software. Each reaction was performed in triplicate, with final calculations resulting from the means of triplicate wells. The ΔΔCq method was used to determine the difference for the mean mRNA expression levels of *TF* between study subjects with different genotypes of rs1361600. For each individual, the relative expression level ΔCq (Cq T – Cq E) of *TF* mRNA was normalized with *GAPDH* and then transformed into a relative quantity using the following formula, where ΔΔCq is for the individual and ΔCq is the calibrator:

$$ \mathrm{Relative}\ \mathrm{quantity} = {2}^{\hbox{--} \Delta \Delta \mathrm{Cq}} $$

The primers for *TF* mRNA were: forward, 5′-CGCCAACTGGTAGACATGG-3′; and reverse, 5′-AACCGGTGCTCTCCACATT-3′. The primers for *GAPDH* were: forward, 5′-TGAAGGTCGGAGTCAACGGATTTGGT-3′; and reverse, 5′-CATGTGGGCCAT GAGGTCCACCAC-3′.

### Serum collection and tissue factor level measurement

Blood samples (2 ml) were collected within 24 hours of meeting the criteria for severe sepsis. Samples were centrifuged at 4°C for 10 minutes at 3,200 rpm within 60 minutes after collection. The serum was then stored at −80°C until use. TF protein levels were determined by the human enzyme-linked immunosorbent assay kit (R&D Systems, Minnesota, USA) according to the manufacturer’s protocol.

### Statistical analysis

The differences in allele and genotype distributions between case and control groups were compared using the chi-square test or Fisher’s exact test when appropriate. The test for association with genotypes used the global genotype test in the 3 × 2 contingency table. Allele frequencies of cases and controls were used to calculate the odds ratio and the 95% confidence interval. Multivariate logistic regression was used to adjust for potential confounding variables including age, gender, history of disease, source of infection, APACHE II and SOFA scores. The Bonferroni method was used to correct for multiple comparisons where applicable. The power analysis was performed using the Genetic Power Calculator web tool. Two-tailed *P* <0.05 was considered statistically significant, whereas corrected *P* < (0.05/number of tests) was considered significant after Bonferroni correction. Thirty-day survival curves were estimated using the Kaplan–Meier method and their comparison related to genotypes was performed with the log-rank test. Continuous variables were described as the mean ± standard deviation. Differences in relative mRNA expression and protein levels of *TF* between the rs1361600AA and GA + GG genotypes were evaluated by Mann–Whitney *U* test. To determine whether an association with rs1361600 genotypes might depend on other potential confounding factors for TF serum levels, we investigated the association of rs1361600 genotypes by adding the polymorphisms to a linear regression model controlling for age, gender and APACHE II scores. The software used for statistical calculations was SPSS 15.0 (SPSS Inc., Chicago, IL, USA) unless specified.

## Results

### Characteristics of the study population

From May 2005 to December 2013, a total of 476 severe sepsis patients (including 126 septic shock patients) and 577 sepsis patients were enrolled in this case–control study. The baseline characteristics and clinical data of all subjects are presented in Table 1. There were no significant differences between the severe sepsis and sepsis groups in age, gender, comorbidity, site of infection and microorganism responsible. However, the length of ICU stay and mean APACHE II and SOFA scores were significantly higher in patients with severe sepsis than those in patients with sepsis (*P* <0.05). The patients with severe sepsis (35.3%) had significantly higher 30-day mortality rate than patients with sepsis (6.9%). According to the 30-day mortality, severe sepsis patients were divided into survivor and nonsurvivor groups. The average age, length of ICU stay and mean APACHE II and SOFA scores in the nonsurvivor group were higher than those in the survivor group (*P* <0.05). However, the gender proportion, comorbidity and site of infection between the nonsurvivor and survivor groups did not show any significant difference (*P* >0.05).

**Table 1:** **Demographic and clinical characteristics of the study subjects**

	**Sepsis patients**	**Severe sepsis patients**	***P*** **value** ^**a**^	**Survivors**	**Nonsurvivors**	***P*** **value** ^**b**^
Number	577	476	NA	308	168	NA
Age	62.6 ± 11.2	66.1 ± 11.5	0.07	62.6 ± 10.8	68.9 ± 13.7	0.04
Sex (male/female)	334/243	275/201	0.56	180/128	98/70	0.98
APACHE II score	10.4 ± 3.3	18.7 ± 4.8	0.007	14.6 ± 2.8	26.2 ± 7.1	0.008
Diabetes	50 (8.7%)	51 (10.7%)	0.26	33 (10.7%)	18 (10.7%)	1.00
Chronic liver disease	11 (1.9%)	17 (3.6%)	0.10	9 (2.9%)	8 (4.8%)	0.30
Chronic renal failure	18 (3.1%)	20 (4.2%)	0.35	12 (3.9%)	8 (4.8%)	0.65
Congestive heart failure	25 (4.3%)	32 (6.7%)	0.09	20 (6.5%)	12 (7.1%)	0.79
Chronic pulmonary disease	35 (6.1%)	42 (8.8%)	0.09	25 (8.1%)	17 (10.1%)	0.46
SOFA score	1.4 ± 0.3	8.1 ± 1.7	<0.001	6.8 ± 1.8	10.5 ± 1.8	0.01
LOS (days)	8.5 ± 2.6	15.3 ± 8.6	0.006	13.5 ± 7.3	15.1 ± 8.9	0.02
Infection insult						
Lung	380 (65.9%)	312 (65.5%)	0.92	206 (66.9%)	106 (63.1%)	0.41
Abdomen	151 (26.2%)	119 (25.0%)	0.67	73 (23.7%)	46 (27.4%)	0.38
Bloodstream	13 (2.3%)	17 (3.6%)	0.20	8(2.6%)	9 (5.4%)	0.12
UTI	11 (1.9%)	12 (2.5%)	0.50	9 (2.9%)	3 (1.8%)	0.45
Others	22 (3.8%)	16 (3.4%)	0.70	12 (3.9%)	4 (2.4%)	0.38
Microbiology positive^c^	225 (39.0%)	195 (41.0%)	0.52	114 (37.0%)	76 (45.2%)	0.08
Gram-positive	96 (42.7%)	75 (38.5%)	0.38	47 (41.2%)	27 (35.5%)	0.43
Gram-negative	98 (43.6%)	81 (41.5%)	0.68	47 (41.2%)	31 (40.8%)	0.95
Fungi	15 (6.7%)	18 (9.2%)	0.33	10 (8.8%)	8 (10.5%)	0.69
Mixed	16 (7.1%)	21 (10.8%)	0.19	10 (8.8%)	10 (13.2%)	0.34

Data presented as mean ± standard deviation or number (%). APACHE, Acute Physiology and Chronic Health Evaluation; LOS, length of ICU stay; NA, not applicable; SOFA, Sequential Organ Failure Assessment; UTI, urinary tract infection. ^a^Sepsis group versus severe sepsis group. ^b^Survivor group versus nonsurvivor group. ^c^Included relevant cultures from tissue sites, sputum, blood, urine, pleural and peritoneal fluid.

### Association analyses of *TF* and *TFPI* polymorphisms with susceptibility to severe sepsis

The distributions of *TF* and *TFPI* SNPs in the patients with sepsis and severe sepsis are presented in Table 2. All tested SNPs conformed to the Hardy–Weinberg equilibrium in both groups (*P* >0.05). We compared the allele and genotype distributions of all SNPs in *TF* and *TFPI* between the sepsis and severe sepsis patients. No significant difference was observed between the two groups in either the unadjusted or adjusted models (*P* >0.05). Assuming the prevalence of 0.01 and using a significance level of 0.05, our study had over 99% power to detect association with rs1361600 in 577 sepsis patients versus 476 severe sepsis patients. These results suggested that the TF and TFPI gene variants were not associated with the susceptibility of severe sepsis in Chinese Han populations.

**Table 2:** **Association analysis of SNPs in** ***TF*** **and** ***TFPI*** **between the sepsis and severe sepsis groups**

**Gene**	**Sepsis**	**Severe sepsis**	**Allelic comparison**				**Genotypic comparison**	
**SNP**	**patients**	**patients**	**P** ^**1**^_**adj**_	**OR** ^**1**^_**adj**_ (**95% CI)**	**P** ^**2**^_**adj**_	**OR** ^**2**^_**adj**_ (**95% CI)**	**P** ^**1**^_**adj**_	**P** ^**2**^_**adj**_
**TF**								
rs1324214			0.13	0.86 (0.71 to 1.05)	0.22	0.89 (0.83 to 1.04)	0.09	0.14
CC	270 (47.8%)	253 (54.2%)						
CT	270 (47.8%)	191 (40.9%)						
TT	25 (4.4%)	23 (4.9%)						
T	810 (71.7%)	697 (74.6%)						
C	320 (28.3%)	237 (25.4%)						
rs762484			0.22	0.86 (0.69 to 1.09)	0.36	0.89 (0.74 to 1.07)	0.07	0.12
TT	374 (66%)	330 (71%)						
TC	185 (32.6%)	124 (26.7%)						
CC	8 (1.4%)	11 (2.4%)						
T	933 (82.3%)	784 (84.3%)						
C	201 (17.7%)	146 (15.7%)						
rs696619			0.31	0.91 (0.76 to 1.09)	0.46	0.93 (0.79 to 1.06)	0.14	0.26
AA	228 (40.7%)	192 (41.5%)						
AG	271 (48.4%)	237 (51.2%)						
GG	61 (10.9%)	34 (7.3%)						
A	727 (64.9%)	621 (67.1%)						
G	393 (35.1%)	305 (32.9%)						
rs3917615			0.87	0.98 (0.80 to 1.21)	0.92	0.98 (0.83 to 1.18)	0.07	0.16
CC	344 (61.3%)	280 (59.5%)						
CT	189 (33.7%)	179 (38%)						
TT	28 (5%)	12 (2.5%)						
C	877 (78.2%)	739 (78.5%)						
T	245 (21.8%)	203 (21.5%)						
rs1361600			0.53	0.94 (0.76 to 1.15)	0.72	0.95 (0.82 to 1.12)	0.07	0.16
AA	342 (60%)	280 (59.6%)						
AG	198 (34.7%)	178 (37.9%)						
GG	30 (5.3%)	12 (2.6%)						
A	882 (77.4%)	738 (78.5%)						
G	258 (22.6%)	202 (21.5%)						
rs958587			0.62	0.95 (0.77 to 1.17)	0.78	0.96 (0.84 to 1.14)	0.06	0.15
CC	342 (60.4%)	279 (59.4%)						
CT	194 (34.3%)	178 (38.0%)						
TT	30 (5.3%)	12 (2.6%)						
C	878 (77.6%)	736 (78.5%)						
T	254 (22.4%)	202 (21.5%)						
rs3917643			1.00	0.22 (0.25 to 6.05)	0.85	0.29 (0.32 to 5.26)	1.00	0.98
AA	570 (99.5%)	468 (99.4%)						
AG	3 (0.5%)	3 (0.6%)						
A	1143 (99.7%)	939 (99.7%)						
G	3 (0.3%)	3 (0.3%)						
rs145977586			1.00	0.22 (0.25 to 6.05)	0.85	0.29 (0.32 to 5.26)	1.00	0.98
GG	570 (99.5%)	468 (99.4%)						
GA	3 (0.5%)	3 (0.6%)						
G	1143 (99.7%)	939 (99.7%)						
A	3 (0.3%)	3 (0.3%)						
**TFPI**								
rs3755248			0.75	0.96 (0.75 to 1.23)	0.67	0.87 (0.71 to 1.18)	0.71	0.62
TT	412 (73%)	342 (73.4%)						
TC	141 (25%)	118 (25.3%)						
CC	11 (2%)	6 (12.9%)						
T	965 (85.5%)	802 (86.1%)						
C	163 (14.5%)	130 (13.9%)						
rs3213739			0.46	1.07 (0.90 to 1.28)	0.32	1.15 (0.82 to 1.32)	0.79	0.56
GG	270 (47.7%)	218 (46.4%)						
GT	156 (27.6%)	127 (27%)						
TT	140 (24.7%)	125 (26.6%)						
G	696 (61.5%)	563 (59.9%)						
T	436 (38.5%)	377 (40.1%)						
rs7594359			0.93	0.99 (0.78 to 1.25)	0.94	0.94 (0.79 to 1.18)	0.12	0.17
CC	389 (69.5%)	317 (68.2%)						
CT	156 (27.9%)	143 (30.8%)						
TT	15 (2.7%)	5 (1.1%)						
C	934 (83.4%)	777 (83.5%)						
T	186 (16.6%)	153 (16.5%)						
rs10931292			0.92	0.99 (0.82 to 1.19)	0.82	0.78 (0.76 to 1.12)	0.14	0.12
TT	255 (44.9%)	222 (47.9%)						
TC	253 (44.5%)	180 (38.9%)						
CC	60 (10.6%)	61 (13.2%)						
T	763 (67.2%)	624 (67.4%)						
C	373 (32.8%)	302 (32.6%)						
rs8176441			0.93	0.99 (0.81 to 1.21)	0.82	0.76 (0.56 to 1.48)	0.10	0.23
TT	300 (52.9%)	259 (55.7%)						
TC	237 (41.8%)	170 (36.6%)						
CC	30 (5.3%)	36 (7.7%)						
T	837 (73.8%)	688 (74%)						
C	297 (26.2%)	242 (26%)						
rs12613071			0.84	1.02 (0.84 to 1.23)	0.75	1.24 (0.86 to 1.38)	0.98	0.95
TT	288 (50.9%)	233 (50.2%)						
TC	224 (39.6%)	186 (40.1%)						
CC	54 (9.5%)	45 (9.7%)						
T	800 (70.7%)	652 (70.3%)						
C	332 (29.3%)	276 (29.7%)						
rs10153820			0.58	0.95 (0.79 to 1.14)	0.62	0.98 (0.81 to 1.12)	0.15	0.36
GG	254 (45.3%)	230 (49.4%)						
GA	251 (44.7%)	181 (38.8%)						
AA	56 (10%)	55 (11.8%)						
G	759 (67.6%)	641 (68.8%)						
A	363 (32.4%)	291 (31.2%)						
rs8176592			0.12	1.24 (0.94 to 1.63)	0.36	1.16 (0.97 to 1.58)	0.31	0.45
TT	461 (80.9%)	363 (77.1%)						
TC	102 (17.9%)	100 (21.2%)						
CC	7 (1.2%)	8 (1.7%)						
T	1024 (89.8%)	826 (87.7%)						
C	116 (10.2%)	116 (12.3%)						
rs2192824			0.79	1.03 (0.83 to 1.27)	0.65	1.13 (0.92 to 1.46)	0.76	0.68
CC	354 (62.7%)	292 (62.7%)						
CT	185 (32.7%)	148 (31.8%)						
TT	26 (4.6%)	26 (5.6%)						
C	893 (79%)	732 (78.5%)						
T	237 (21%)	200 (21.5%)						

Data presented as number (%) of subjects. *P* was determined using the chi-square test. P_adj_ and OR_adj_ came from multivariate logistic regression. *P* <0.0029 (0.05/17) was considered statistically significant after Bonferroni correction. CI, confidence interval; OR, odds ratio; SNP, single nucleotide polymorphism; TF, tissue factor; TFPI, tissue factor pathway inhibitor.

### Association analyses of TF and TFPI polymorphisms with outcome of severe sepsis

**Figure 1:**
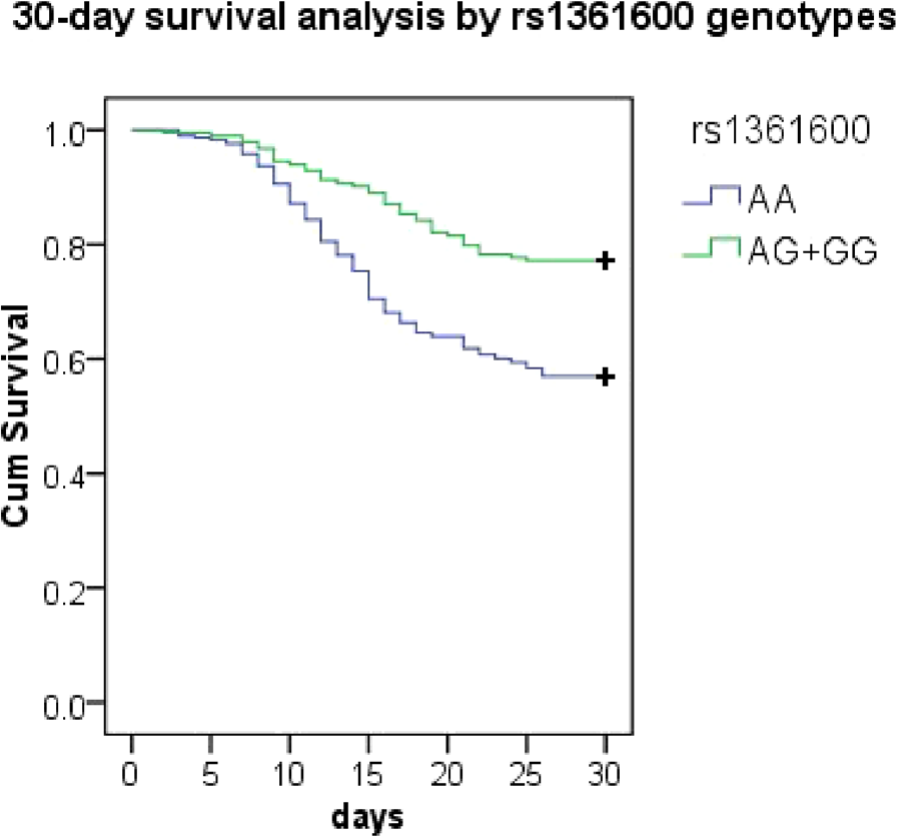
**Kaplan–Meier survival analysis of patients with severe sepsis according to the genotypes of rs1361600.** Differences between groups were compared by log-rank analysis. Kaplan–Meier 30-day survival curves showed strong significant differences between the subjects who carried the AA genotype and the subjects who carried the AG + GG genotypes (*P* = 5.92 × 10^-5^).

We next investigated the association between all tested SNPs and 30-day mortality of patients with severe sepsis. Three SNPs (rs1361600 (−603A/G), rs3917615 (−1322C/T) and rs958587 (−1812C/T)) in the TF gene promoter region were in complete linkage disequilibrium. The –603G/–1322 T/–1812 T haplotype, presently defined as –603G (rs1361600G), was significantly associated with lower mortality at 30 days. As shown in Table 3, the minor allele rs1361600G frequency was significantly more frequent in survivors compared with nonsurvivors (*P* = 4.91 × 10^-5^, odds ratio = 0.48, 95% confidence interval = 0.33 to 0.69), even after Bonferroni correction for multiple comparisons (*P* = 8.35 × 10^-4^ corrected for 17 SNPs tested). Furthermore, using age, APACHE II and SOFA scores as covariates, multiple logistic regression analysis showed that the minor allele G was still significantly associated with protection from severe sepsis fatal outcome (adjusted *P* = 4.52 × 10^-4^, adjusted odds ratio = 0.57, 95% confidence interval = 0.41 to 0.74). The genotype distribution of rs1361600 was also significantly different between the survivor and nonsurvivor groups (*P* = 2.42 × 10^-5^), and the difference remained significant after adjustment for age, APACHE II and SOFA scores in multiple logistic regression analysis (adjusted *P* = 6.51 × 10^-4^) and for multiple comparisons (*P* = 4.11 × 10^-4^ corrected for 17 SNPs tested). We then conducted a Kaplan–Meier survival analysis of different rs1361600 genotypes in patients with severe sepsis at 30 days (Figure 1). Kaplan–Meier survival curves showed strong significant differences between the subjects who carried the AA genotype and the subjects who carried AG + GG genotypes (log-rank test, *P* =5.92 × 10^-5^). However, the SNPs in *TFPI* were not associated with the outcome for severe sepsis patients (*P* >0.05) (Table 3).

**Table 3:** **Association analysis of SNPs in** ***TF*** **and** ***TFPI*** **between the survivor and nonsurvivor groups**

**Gene**	**Survivor**	**Nonsurvivor**	**Allelic comparison**				**Genotypic comparison**	
**SNP**			**P** ^**1**^_**adj**_	**OR** ^**1**^_**adj**_ (**95% CI)**	**P** ^**2**^_**adj**_	**OR** ^**2**^_**adj**_ (**95% CI)**	**P** ^**1**^_**adj**_	**P** ^**2**^_**adj**_
**TF**								
rs1324214			0.06	0.73 (0.53 to 1.01)	0.12	0.78 (0.56 to 1.16)	0.13	0.24
CC	165 (55%)	103 (62.8%)						
CT	118 (39.3%)	57 (34.8%)						
TT	17 (5.7%)	4 (2.4%)						
T	448 (74.7%)	263 (80.2%)						
C	152 (25.3%)	65 (19.8%)						
rs762484			0.13	0.75 (0.51 to 1.09)	0.26	0.78 (0.82 to 1.07)	0.32	0.38
TT	208 (68.6%)	124 (74.7%)						
TC	85 (28%)	39 (23.5%)						
CC	10 (3.3%)	3 (1.8%)						
T	501 (82.7%)	287 (86.4%)						
C	105 (17.3%)	45 (13.6%)						
rs696619			0.23	0.84 (0.63 to 1.12)	0.38	0.86 (0.73 to 1.09)	0.41	0.52
AA	122 (40.4%)	75 (45.7%)						
AG	156 (51.7%)	80 (48.8%)						
GG	24 (7.9%)	9 (5.5%)						
A	400 (66.2%)	230 (70.1%)						
G	204 (33.8%)	98 (29.9%)						
rs3917615			0.00015	0.50 (0.35 to 0.72)	0.0018	0.69 (0.45 to 0.82)	0.00014	0.0017
CC	167 (55.1%)	123 (74.5%)						
CT	126 (41.6%)	39 (23.6%)						
TT	10 (3.3%)	3 (1.8%)						
C	460 (75.9%)	285 (86.4%)						
T	146 (24.1%)	445 (13.6%)						
rs1361600			0.000049	0.48 (0.33 to 0.69)	0.00045	0.57 (0.41 to 0.74)	0.000024	0.00065
AA	164 (53.6%)	124 (74.7%)						
AG	130 (42.5%)	38 (22.9%)						
GG	12 (3.9%)	4 (2.4%)						
A	458 (74.8%)	286 (86.1%)						
G	154 (25.2%)	46 (13.9%)						
rs958587			0.0001	0.50 (0.34 to 0.71)	0.0015	0.67 (0.43 to 0.79)	0.000094	0.0016
CC	164 (53.9%)	122 (73.9%)						
CT	130 (42.8%)	40 (24.2%)						
TT	10 (3.3%)	3 (1.8%)						
C	458 (75.3%)	284 (86%)						
T	150 (24.7%)	46 (14%)						
rs3917643			0.62	1.84 (0.26 to 13.10)	0.78	1.48 (0.76 to 14.07)	0.62	0.78
AA	304 (99.3%)	165 (98.8%)						
AG	2 (0.7%)	2 (1.2%)						
A	610 (99.7%)	332 (99.4%)						
G	2 (0.3%)	2 (0.6%)						
rs145977586			1.00	0.92 (0.08 to 10.14)	0.95	0.93 (0.12 to 12.76)	1.00	0.95
GG	304 (99.3%)	166 (99.4%)						
GA	2 (0.7%)	1 (0.6%)						
G	610 (99.7%)	333 (99.7%)						
A	2 (0.3%)	1 (0.3%)						
**TFPI**								
rs3755248			0.75	0.94 (0.63 to 1.39)	0.62	0.84 (0.56 to 1.23)	0.13	0.11
TT	222 (72.8%)	126 (76.4%)						
TC	80 (26.2%)	34 (20.6%)						
CC	3 (1%)	5 (3%)						
T	524 (85.9%)	286 (86.7%)						
C	86 (14.1%)	44 (13.3%)						
rs3213739			0.16	0.82 (0.62 to 1.08)	0.25	0.85 (0.72 to 1.04)	0.44	0.52
GG	135 (44.7%)	84 (50.9%)						
GT	84 (27.8%)	41 (24.8%)						
TT	83 (27.5%)	40 (24.2%)						
G	354 (58.6%)	209 (63.3%)						
T	250 (41.4%)	121 (36.7%)						
rs7594359			0.18	0.78 (0.54 to 1.12)	0.12	0.71 (0.46 to 1.09)	0.23	0.18
CC	197 (64.8%)	120 (71.9%)						
CT	104 (34.2%)	45 (26.9%)						
TT	3 (1%)	2 (1.2%)						
C	498 (81.9%)	285 (85.3%)						
T	110 (18.1%)	49 (14.7%)						
rs10931292			0.44	0.89 (0.67 to 1.19)	0.47	0.91 (0.81 to 1.13)	0.38	0.49
TT	142 (47%)	87 (52.7%)						
TC	122 (40.4%)	56 (33.9%)						
CC	38 (12.6%)	22 (13.3%)						
T	406 (67.2%)	230 (69.7%)						
C	198 (32.8%)	100 (30.3%)						
rs8176441			0.87	1.03 (0.75 to 1.40)	0.95	0.92 (0.61 to 1.32)	0.06	0.17
TT	185 (60.9%)	90 (55.6%)						
TC	89 (29.3%)	63 (38.9%)						
CC	30 (9.8%)	9 (5.5%)						
T	459 (75.5%)	243 (75%)						
C	149 (24.5%)	81 (25%)						
rs12613071			0.91	1.02 (0.76 to 1.37)	0.72	1.12 (0.83 to 1.58)	0.32	0.21
TT	150 (50.2%)	86 (52.8%)						
TC	124 (41.5%)	58 (35.6%)						
CC	25 (8.4%)	19 (11.7%)						
T	424 (70.9%)	230 (70.6%)						
C	174 (29.1%)	96 (29.4%)						
rs10153820			0.20	1.21 (0.91 to 1.61)	0.45	1.15 (0.82 to 1.46)	0.25	0.52
CC	161 (53.5%)	76 (45.8%)						
CT	105 (34.9%)	70 (42.2%)						
TT	35 (11.6%)	20 (12%)						
C	427 (70.9%)	222 (66.9%)						
T	175 (29.1%)	110 (33.1%)						
rs8176592			0.35	0.81 (0.52 to 1.26)	0.62	0.84 (0.71 to 1.19)	0.34	0.58
TT	234 (77%)	133 (81.6%)						
TC	68 (22.4%)	28 (17.2%)						
CC	2 (0.6%)	2 (1.2%)						
T	536 (88.2%)	294 (90.2%)						
C	72 (11.8%)	32 (9.8%)						
rs2192824			0.48	0.89 (0.64 to 1.24)	0.52	0.91 (0.72 to 1.19)	0.38	0.41
CC	190 (62.9%)	106 (63.9%)						
CT	94 (31.1%)	55 (33.1%)						
TT	18 (6%)	5 (3%)						
C	474 (78.5%)	267 (80.4%)						
T	130 (21.5%)	65 (19.6%)						

Data presented as number (%) of subjects. *P* was determined using the chi-square test. P_adj_ and OR_adj_ came from multivariate logistic regression. *P* <0.0029 (0.05/17) was considered statistically significant after Bonferroni correction. CI, confidence interval; OR, odds ratio; SNP, single nucleotide polymorphism; TF, tissue factor; TFPI, tissue factor pathway inhibitor.

### Association analyses of tissue factor mRNA expression levels with rs1361600 genotypes

**Figure 2:**
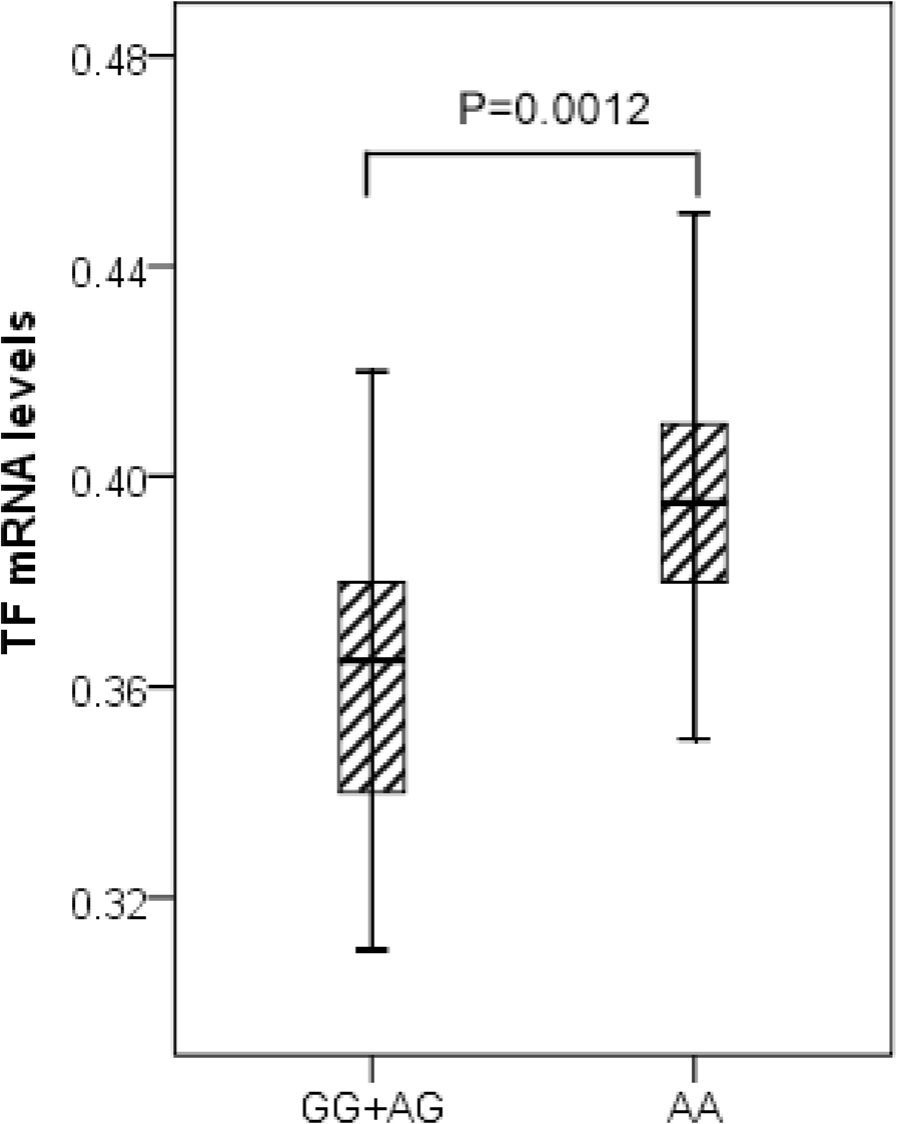
**Association of tissue factor mRNA levels and rs1361600 genotypes in healthy volunteers.** The tissue factor (*TF*) mRNA levels of peripheral blood mononuclear cells expressed as the median, interquartile range and extremes. The *TF* mRNA levels were significantly different between individuals with the AG + GG and the AA genotypes under the lipopolysaccharide-stimulated condition (*P* = 0.0012).

To evaluate the association between the rs1361600 genotype and *TF* mRNA levels in PBMCs, we selected 45 subjects with the rs1361600AA genotype, 42 subjects with the AG genotype and three subjects with the GG genotype, who were matched for age and sex. As shown in Figure 2, the *TF* mRNA expression in PBMCs was significantly lower in the subjects who carried AG + GG genotypes compared with the subjects who carried AA genotype after stimulation with LPS for 6 hours (*P* = 0.0012).

### Association analyses of tissue factor plasma levels with rs1361600 genotypes

**Figure 3:**
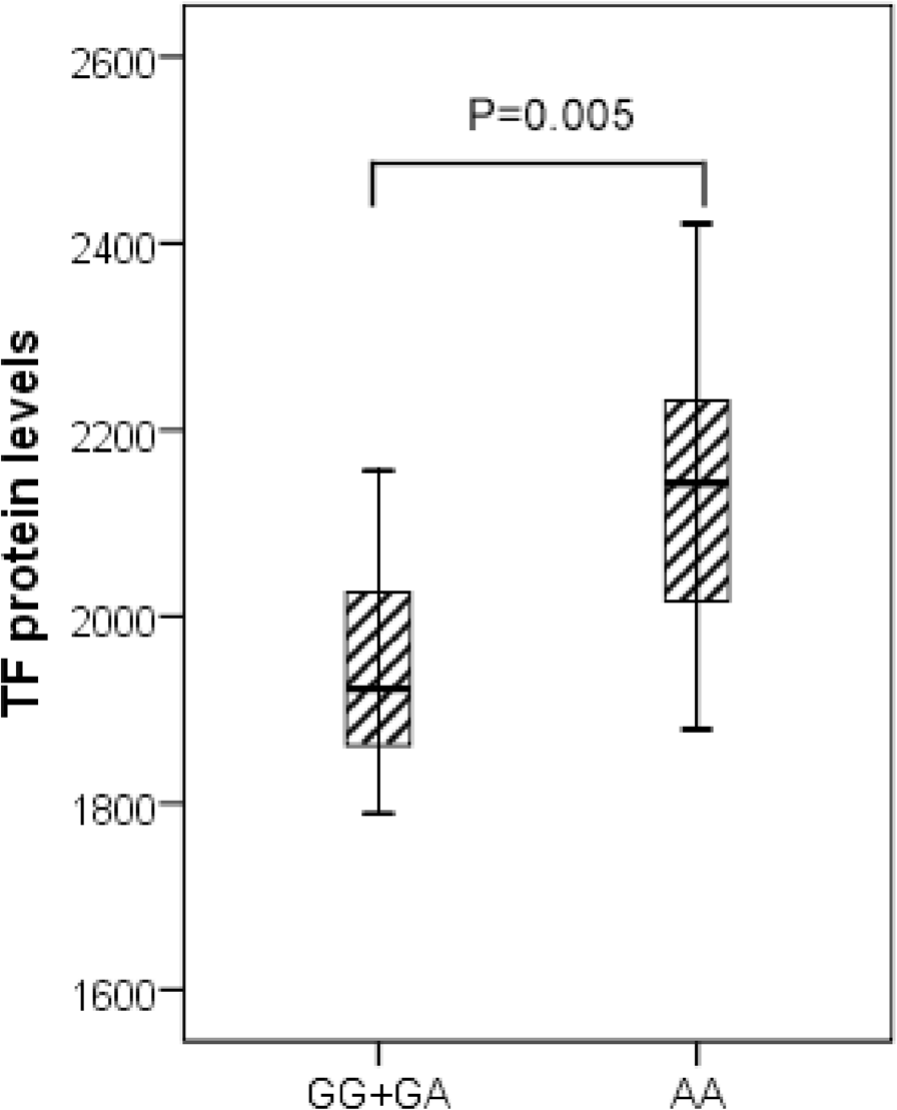
**Association of tissue factor levels and rs1361600 genotypes in severe sepsis patients.** Tissue factor (TF) serum levels in severe sepsis patients were expressed as the median, interquartile range and extremes. The TF levels were significantly different between individuals with AG + GG and AA genotypes (*P* = 0.005). The difference remained significant (adjusted *P* = 0.02) after adjustment for age, gender, Acute Physiology and Chronic Health Evaluation II and Sequential Organ Failure Assessment scores in a linear regression model.

To assess the influence of rs1361600 on plasma TF protein levels, TF antigen of plasma was measured in 118 severe sepsis patients, including 60 patients with the rs1361600AA genotype, 55 patients with the AG genotype and three patients with the GG genotype. Our results showed that rs1361600AG + GG genotypes were associated with lower TF serum concentrations on the first day of severe sepsis. As shown in Figure 3, the patients with AG + GG genotypes had lower plasma concentrations of TF than AA subjects (1,945.6 ± 168.5 pg/ml vs. 2,124.3 ± 273.6 pg/ml, *P* =0.005). To control confounding variables, we used the possible confounding factors (age, APACHE II and SOFA scores) as covariates in a linear regression model and found that the rs1361600 genotypes remained association with TF serum concentration (adjusted *P* = 0.02). Moreover, we found that the plasma level of TF on the first day of severe sepsis was higher in patients who died than in survivors (3,036.3 ± 423.8 pg/ml vs. 1,650.7 ± 316.7 pg/ml, adjusted *P* =0.01).

## Discussion

To the best of our knowledge, this is the first study testing the association between severe sepsis and the polymorphisms of *TF* and *TFPI* in Chinese Han populations. We found that the *TF* rs1361600 (−603)G allele, tagging the –603G/–1322 T/–1812 T haplotype in the promoter of *TF*, is associated with the clinical outcome in patients with severe sepsis. Further evidence of the role of the rs1361600G allele in the pathogenesis of severe sepsis is provided by the observed significant association of this allele with decreased *TF* mRNA transcription *in vitro*. Moreover, severe sepsis patients with the rs1361600G allele showed lower serum TF levels. In addition, we found that the plasma levels of TF in patients with severe sepsis are positively related to poor outcome.

Over the last decade, the cross-talk between inflammation and coagulation systems emerged as a major mechanism controlling the host response to the invading microorganisms, and poor regulation of this mechanism was held responsible for the occurrence of multiple organ failure and eventually death in patients with severe sepsis [3]. Recent studies indicated that most patients with severe sepsis had coagulation abnormalities and the higher levels of TF, the main regulator of coagulation process, were closely related with the severity and outcome of sepsis [4]. Our results showed that the plasma levels of TF are significantly higher in nonsurviving septic patients than in surviving septic patients. Exaggerated and uncontrolled coagulation response to the infecting pathogens may lead to microvascular thrombosis and multiple organ dysfunction syndrome [25,26]. In models of endotoxemia, mice heterozygous for mutations in antithrombin III and protein C exhibited increased tissue fibrin deposition and mortality compared with wild-type controls, presumably due to reduced levels of antithrombin III and active protein C [27]. Pawlinski and colleagues found that reduced expression of TF resulted in diminished LPS-induced coagulation, inflammation and mortality [5]. All of these studies illustrate that downregulation of any protein involved in hemostasis had a dramatic effect on the whole immune response and influenced sepsis-related mortality. Among several molecules involved in the coagulation complex, TF had an important role in the initiation of coagulation and in thrombin activation at the site of inflammation [10]. TF is a 47 kDa membrane-bound protein that is expressed in a wide variety of tissues, including circulating blood cells, vascular endothelial cells and hematopoietic cells. TF is considered responsible for the initiation of coagulation in sepsis [28]. Apart from its function in the coagulation system, TF attracted considerable attention as a potential mediator in intracellular signaling of established inflammatory pathways, functioning as an intermediate for factor VIIa-induced activation of mitogen-activated protein kinases and calcium signaling. These effects were probably mediated by binding of TF to protease activated receptor-1 and protease activated receptor-2 on various cell types [29,30].

The TF gene is located at chromosome 1p21.3. The involvement of TF in pathological processes may be modulated by genetic factors influencing TF expression and activity. Several common variants within the TF gene have been described [31], such as two haplotypes formed by the four completely concordant promoter polymorphisms C-1812 T (rs958587), C-1322 T (rs3761955), Del-1208Ins (rs ID not available) and A-603G (rs1361600). Although not in a fully conclusive manner, these promoter haplotypes were associated with TF expression levels and/or activity as well as to thrombotic and atherosclerosis risk in several studies [18,19,21]. However, to our knowledge, until now no study has investigated the role of genetic variation of *TF* in the risk of severe sepsis. In the present study, we found that severe sepsis patients with the *TF*-rs1361600G allele had a higher rate of survival at 30 days. The association between severe sepsis death and rs1361600 may be partly explained by differentiated expression of *TF* mRNA in monocytes stimulated with LPS from donors carrying either the rs1361600 A or G allele. The G allele was associated with a lower expression of *TF* mRNA in LPS-induced samples. Our findings are in accordance with the result from Terry and colleagues that the −1208I allele, in perfect linkage disequilibrium with rs1361600G, was associated with lower expression and activity of *TF* in human umbilical vein endothelial cells induced by interleukin-1 or phorbol 12-myristate 13-acetate [32]. Moreover, we found that circulating plasma levels of TF in severe sepsis patients with the AG + GG genotype of rs1361600 were significantly lower than those with the AA genotype. As TF plays a pivotal role in the pathogenesis of severe sepsis in response to infection, it is reasonable to assume that patients with the rs361600A allele might produce a higher amount of TF, and thereby enhance vascular TF expression to initiate coagulation and promote microvascular thrombosis, which contributes to organ injury during severe sepsis.

Our study has several clear strengths. First, mild sepsis patients served as controls in our study. Such controls were preferable to healthy individuals since a proportion of healthy subjects might develop severe sepsis under the stimulus of infection. Second, to minimize racial admixture, we focused on central Han Chinese patients, which could be regarded as one single homogeneous population. Of note, there were two limitations to the current study. First, although we had adequate power to detect rs1361600 association with severe sepsis using the current data, independent samples were still needed to validate the associations. Second, we did not resequence the TF and TFPI genes. Instead, only 17 SNPs including two functional SNPs were genotyped in our study, which was far from comprehensive. Some important SNPs might possibly therefore have been missed or the observed association might be due to other polymorphisms in linkage disequilibrium with the studied ones.

## Conclusion

In summary, our results indicate that genetic variations in the promoter region of *TF* are associated with the outcome of severe sepsis in Chinese Han population through influencing the *TF* mRNA expression. These data support the concept that genetic variation in the hemostatic gene plays an important role in the development and outcome of severe sepsis. Identifying these genetic factors might, in the future, help to choose appropriate therapy for patients at different risk.

## Key messages

- Individuals carrying the G allele of rs1361600 in the *TF* gene have a lower risk for death of severe sepsis in Chinese Han population.
- SNP rs1361600 influences the expression of *TF* mRNA and the protein production of TF in patients with severe sepsis.

## Additional files

Additional file 1: Table S1. Presenting the definition of sepsis, severe sepsis and septic shock. Additional file 2: Table S2. Presenting the location and characterization of all selected SNPs within the *TF* and *TFPI* genes. Additional file 3: Table S3. Presenting the primers of 17 tag SNPs in the *TF* and *TFPI* genes.

## Abbreviations

APACHE: Acute Physiology and Chronic Health Evaluation
LPS: lipopolysaccharide
PBMC: peripheral blood mononuclear cell
SNP: single nucleotide polymorphism
SOFA: Sequential Organ Failure Assessment
TF: tissue factor
TFPI: tissue factor pathway inhibitor
