# Supplementary material for: Genetic variation in the tissue factor gene is associated with clinical outcome in severe sepsis patients
Source: Crit Care. 2014 Nov 17;18(6):631. doi: 10.1186/s13054-014-0631-9 (PMC4271362; doi:10.1186/s13054-014-0631-9)
Supplement: Additional file 1: Table S1. — Presenting the definition of sepsis, severe sepsis and septic shock. [file 13054_2014_631_MOESM1_ESM.doc]

**Supplementary table 1. The definition of sepsis, severe sepsis and septic shock**

|  | Definition |
| --- | --- |
| Sepsis | Known or suspected source of systemic infection plus at least two of the following: a) temperature > 38°C or < 36°C; b) heart rate > 90 beats/min; c) respiratory rate > 20 breaths/min or PaCO_2_ < 32 mmHg; d) WBC count > 12,000/mm^3^, < 4000/mm^3^, or > 10% bandemia. |
| Severe sepsis | Sepsis associated with organ dysfunction, hypoperfusion, or hypotension. Hypoperfusion and perfusion abnormalities may include, but are not limited to lactic acidosis, oliguria, or an acute alteration in mental status. Organ dysfunction parameters: a) arterial hypoxemia (PaO_2_/FiO_2_ < 300); b) acute oliguria (urine output <0.5 ml kg^−1^ h^−1^ or 45 mM/l for at least 2 h); c) creatinine increase ≥0.5 mg/dl; d) coagulation abnormalities (international normalized ratio >1.5 or activated partial thromboplastin time >60 s); e) ileus (absent bowel sounds); f) thrombocytopenia (platelet count <100,000/μl); g) hyperbilirubinemia (plasma total bilirubin > 4 mg/dl or 70 mmol/l). |
| Septic shock | Fulfill requirements for sepsis plus one of the following: a) Systolic arterial pressure below 90 mmHg, mean arterial pressure lower than 60, or a reduction in systolic blood pressure of more than 40 mmHg from baseline, despite adequate volume resuscitation, in the absence of other cause of hypotension.; b) need for vasopressors to maintain systolic arterial pressure ≥ 90 mmHg, mean arterial pressure ≥ 60 mmHg or within 40 mmHg of baseline. |
