# Supplementary material for: Genetic variation in the tissue factor gene is associated with clinical outcome in severe sepsis patients
Source: Crit Care. 2014 Nov 17;18(6):631. doi: 10.1186/s13054-014-0631-9 (PMC4271362; doi:10.1186/s13054-014-0631-9)
Supplement: Additional file 2: Table S2. — Presenting the location and characterization of all selected SNPs within the TF and TFPI genes. [file 13054_2014_631_MOESM2_ESM.doc]

Table S2: Characteristics of all tested SNPs in the TF and TFPI gene

| Gene | SNP | Location | Major/minor allele | HWE P value |
| --- | --- | --- | --- | --- |
| TF | rs1324214 | intron | C/T | 0.36 |
| TF | rs762484 | intron | T/C | 0.17 |
| TF | rs696619 | intron | A/G | 0.11 |
| TF | rs3917615 | intron | C/T | 0.69 |
| TF | rs3917643 | intron | A/G | 1.00 |
| TF | rs145977586 | exon | G/A | 1.00 |
| TF | rs1361600 | 5'UTR | A/G | 0.71 |
| TF | rs958587 | 5'UTR | C/T | 0.78 |
| TFPI | rs3755248 | intron | T/C | 1.00 |
| TFPI | rs3213739 | intron | G/T | 0.56 |
| TFPI | rs7594359 | intron | C/T | 0.52 |
| TFPI | rs10931292 | 5'UTR | T/C | 0.44 |
| TFPI | rs8176441 | intron | T/C | 1.00 |
| TFPI | rs12613071 | intron | T/C | 0.27 |
| TFPI | rs10153820 | 5'UTR | C/T | 0.59 |
| TFPI | rs8176592 | intron | T/C | 1.00 |
| TFPI | rs2192824 | intron | C/T | 0.37 |

SNP, single nucleotide polymorphism; HWE, Hardy-Weinberg equilibrium; UTR, untranslated region
