# Supplementary material for: Genetic variation in the tissue factor gene is associated with clinical outcome in severe sepsis patients
Source: Crit Care. 2014 Nov 17;18(6):631. doi: 10.1186/s13054-014-0631-9 (PMC4271362; doi:10.1186/s13054-014-0631-9)
Supplement: Additional file 3: Table S3. — Presenting the primers of 17 tag SNPs in the TF and TFPI genes. [file 13054_2014_631_MOESM3_ESM.docx]

**Supplementary table 3 The primers of seventeen tagSNPs in the TF and TFPI genes**

| Gene | SNP | Oligo type-mode | Sequence |
| --- | --- | --- | --- |
| TF | rs1324214 | PCRU | ATATGTTGGGAGCTCAGGC |
|  |  | PCRL | AGGCTTGCAGCTTCCTGC |
|  |  | SNPU | AGATAGAGTCGATGCCAGCTCATTGTGCGAGGAACAGTCCCTAAC |
| TF | rs762484 | PCRU | CTGATGGAAAAGCTCCCAG |
|  |  | PCRL | TGATCTGCAAACAGCAGAG |
|  |  | SNPU | GTGATTCTGTACGTGTCGCCCAGCAGCAAGAGATTGTTCAGTCCA |
| TF | rs696619 | PCRU | TTTAGATTGAACATGCTGAATTATC |
|  |  | PCRL | TGGCAGAGCAGAAATACTCTG |
|  |  | SNPU | AGATAGAGTCGATGCCAGCTGGGGACAGCTGCCAGTCAGAGTTGA |
| TF | rs3917615 | PCRU | TTGTAAAAACAAGGCAAATTACAG |
|  |  | PCRL | ATCCTTCTGCTTCGGCCT |
|  |  | SNPU | GACCTGGGTGTCGATACCTAACTCAAACATGCATCCCAAGTGCTC |
| TF | rs3917643 | PCRU | ATAGACTGCTTCCTGGCTGT |
|  |  | PCRL | CTGGGCAACATAGCAGGA |
|  |  | SNPL | GCGGTAGGTTCCCGACATATACAAAACAATGTTTAAAAAAAAGCA |
| TF | rs145977586 | PCRU | AGTTCTCCTTCCAGCTCTGC |
|  |  | PCRL | CAGAAATATTCTACATCATTGGAGC |
|  |  | SNPU | AGAGCGAGTGACGCATACTAATATAGCCAGGATGATGACAAGGAT |
| TF | rs1361600 | PCRU | TTTCTCCTGTGCGACCCG |
|  |  | PCRL | AGCGAAGTAAACGTGTGGC |
|  |  | SNPU | ACGCACGTCCACGGTGATTTAGGGCCCCGCGAGGTGGGCAGGCCA |
| TF | rs958587 | PCRU | TTTTGTATATCCATTAAAATAGACAGGA |
|  |  | PCRL | AAATATTATTTACCACGTTTTCTTCCT |
|  |  | SNPU | AGCGATCTGCGAGACCGTATTTCTTCAAGATTTAAAAATGCTAAA |
| TFPI | rs3755248 | PCRU | AATCTGAGATGACAAGGGCA |
|  |  | PCRL | GTCACACTGAGAGAAAATAAAGC |
|  |  | SNPU | CGACTGTAGGTGCGTAACTCTGGTGAGCTGAGCACCACCAGGTAT |
| TFPI | rs3213739 | PCRU | ACATAATCAAAAGCTTACTTCAAAG |
|  |  | PCRL | AAATACTGAGATTCAAATTGTTTAAGAC |
|  |  | SNPU | GCGGTAGGTTCCCGACATATATACTTTAAAATAGATTAGGAAAAT |
| TFPI | rs7594359 | PCRU | ATGCTACCTGTGCTGTGGT |
|  |  | PCRL | TAATAGCTCTTTTGTGAAAGCAAA |
|  |  | SNPU | AGCGATCTGCGAGACCGTATTTTCCTATCAGCAGCCAAATATAAC |
| TFPI | rs10931292 | PCRU1 | AATGCTTTTTTCCACCTAGAGT |
|  |  | PCRU2 | AATGCTTTTTTCCCCCTAGAGT |
|  |  | PCRL | GTGAAACAAATGAAATAACTTGAAGA |
|  |  | SNPL | CGTGCCGCTCGTGATAGAATGAAGAAGAAACAAACTGCAAAAAAG |
| TFPI | rs8176441 | PCRU | TTTGTTAACTTAGAATAACATATGACAGAGA |
|  |  | PCRL | TTAAAGATTTGATCAACATTCTTACTTG |
|  |  | SNPU | GGATGGCGTTCCGTCCTATTGTTTCAGCACCAAACACAATTGTCA |
| TFPI | rs12613071 | PCRU | TTCAGTGAGGTTGCCTTAAAC |
|  |  | PCRL | TTGAAACCCATAGACAGCTACAG |
|  |  | SNPU | ACGCACGTCCACGGTGATTTCAAATTCCTGACCTTTTAATTTATT |
| TFPI | rs10153820 | PCRU | ATTTGTTTCACCCACTGTCAA |
|  |  | PCRL | GGCTCAAAGCCTGACACC |
|  |  | SNPU | AGGGTCTCTACGCTGACGATTACCGTTGGAGGTCTCTCTTAGTGA |
| TFPI | rs8176592 | PCRU | GGACCGTGAAATTCTAAAA |
|  |  | PCRL | TTTTACATACACATGCAACAACATT |
|  |  | SNPU | CGTGCCGCTCGTGATAGAATCAATCAGGAAAACATGGTAAGCCAT |
| TFPI | rs2192824 | PCRU | AAAATTGCACATGGAAATGG |
|  |  | PCRL | CAAACACATTCAGTGCATATATTTATTA |
|  |  | SNPU | GGATGGCGTTCCGTCCTATTTACATACTTCGCCATATTGATCAGC |

SNP, single nucleotide polymorphism

Note: Primer design for PCR and single base extension (SBE) was performed with Beckman Coulter Autoprimer software. PCR reactions in the first step were composed of PCR primers (PCRU and PCRL) at a final concentration of 50 nM, 0.2 U of enzyme HotStar Taq DNA Polymerase (QIAGEN), and 10 ng of genomic DNA per reaction. PCR products were used as template for the single base extension (SBE). SBE was performed using SBE primers (SNPU&SNPL) and reagents specific to the SNPstream platform. SBE reaction products were hybridized to Beckman array plates and scanned by the SNPstream.
